# EVALUATION OF FALL RISK PREVENTION EDUCATION THROUGH AGE-FRIENDLY PARTNERSHIPS

**DOI:** 10.1093/geroni/igac059.1844

**Published:** 2022-12-20

**Authors:** Solymar Rivera-Torres, Jennifer Severance

**Affiliations:** University of North Texas, Denton, Krum, Texas, United States; UNTHSC-TCOM, Fort Worth, Texas, United States

## Abstract

Falls in the home and community environments are the leading cause of injuries and long-term disabilities for the aging population. This study examines the outcomes of a partnership among an academic institution, government agency, community nonprofit, and emergency services organization to expand access to a fall prevention training program by targeting delivery in postal codes identified as underserved with high rates of falls emergencies (hot spots) and non-high rates (non-hot spots). A total of 354 adults aged 50 and older participated in a fall prevention education program, with 188 (53%) participants completing at least five sessions (completers), of which 35% resided in hot spot areas. Descriptive statistics for frequency, percentage, mean, and standard deviations values were calculated for demographic variables. A paired t-test analysis was conducted to compare initial and final scores for self-assessed general health and fall efficacy programs. The paired sample t-test statistics revealed significant improvements in fall efficacy for completers in hot spots (t= -6.23; p < 0.001) and non-hot spots areas (t= - 11.17; p < 0.001). No statistical differences (p > 0.05) were observed between the initial and final scores of the self-assessed general health for completers in hot spots and non-hot spot areas. Cross-sector collaboration to deliver targeted falls prevention training at various community locations can effectively reach underserved, at-risk older adults, although additional retention strategies must be considered. In conclusion, identifying at-risk older adults to mobilize partnerships, limited resources can be allocated towards improving retention and program outcomes of community-based fall prevention education.

